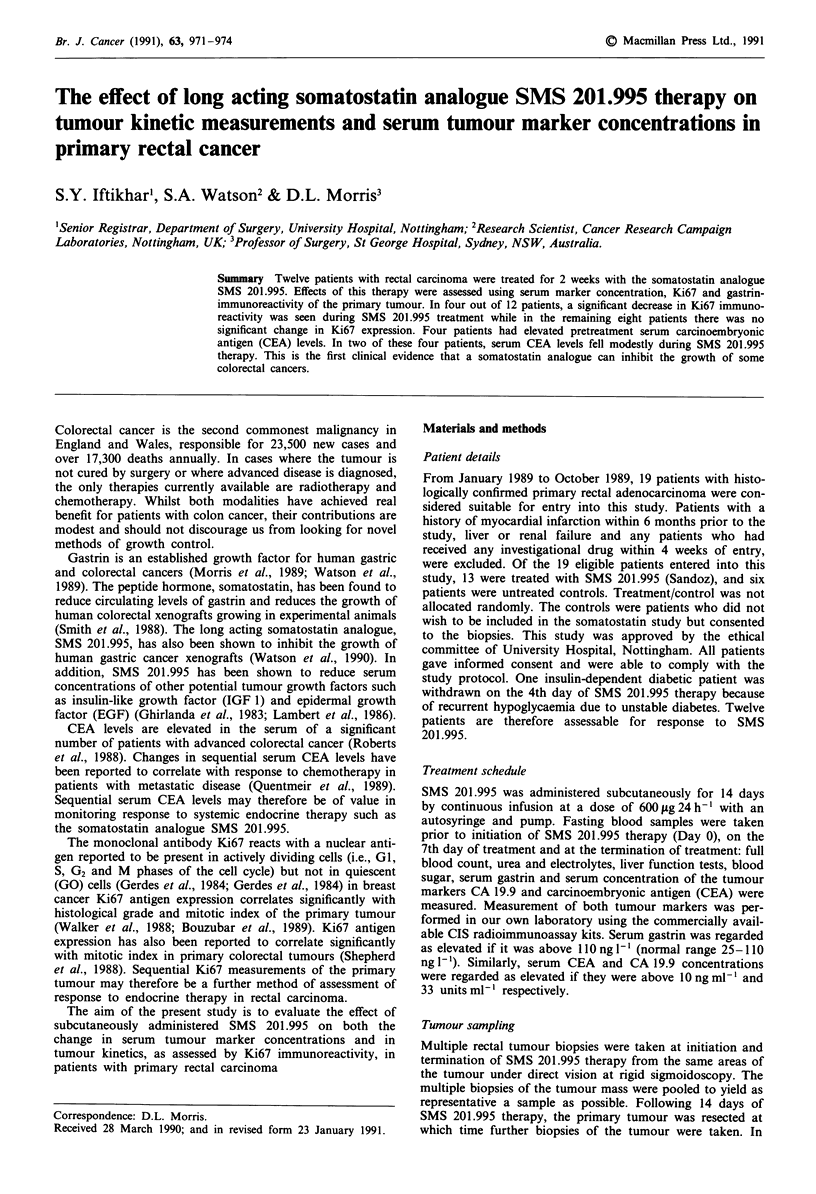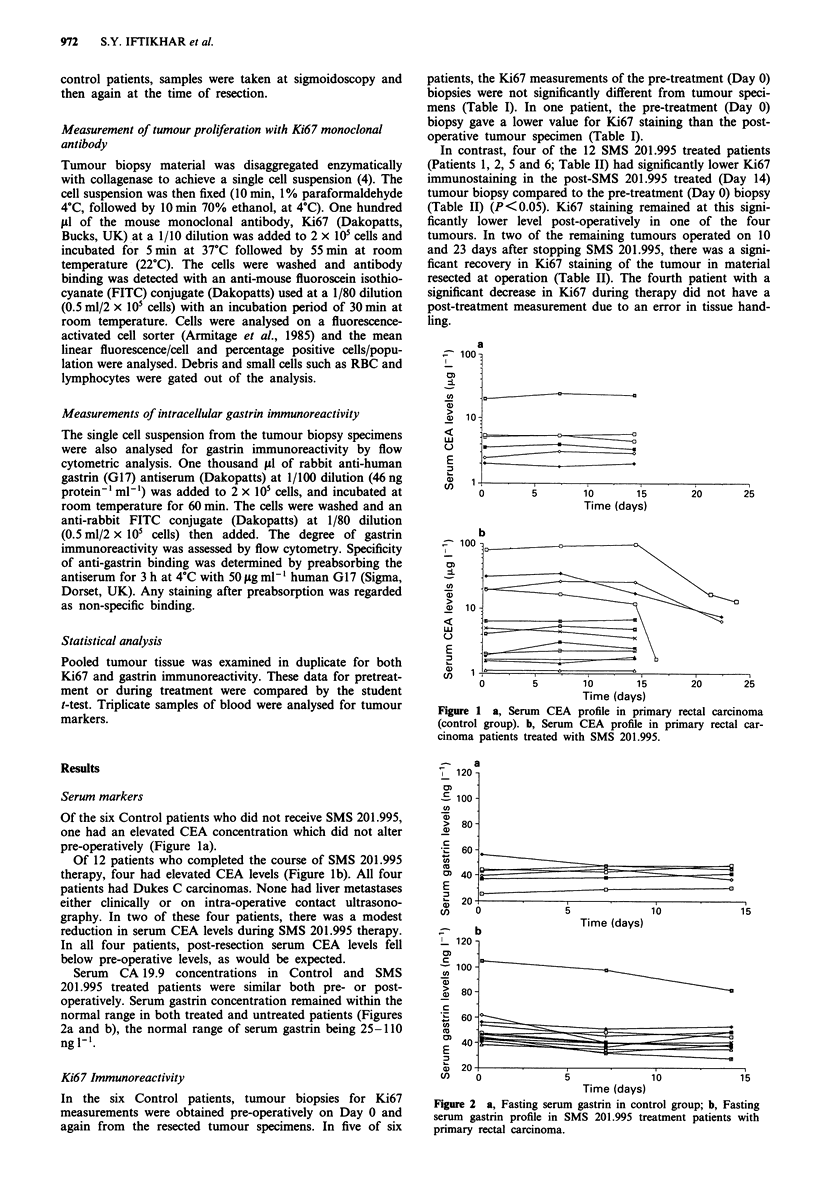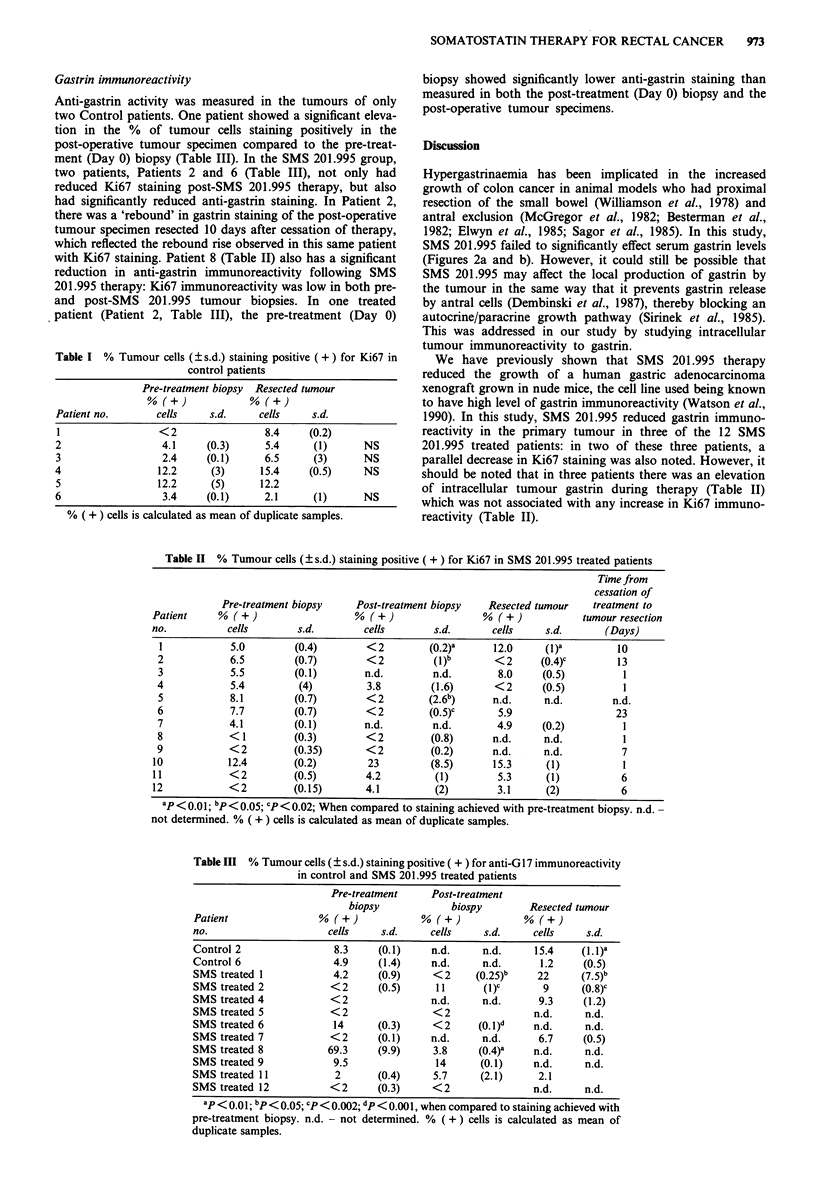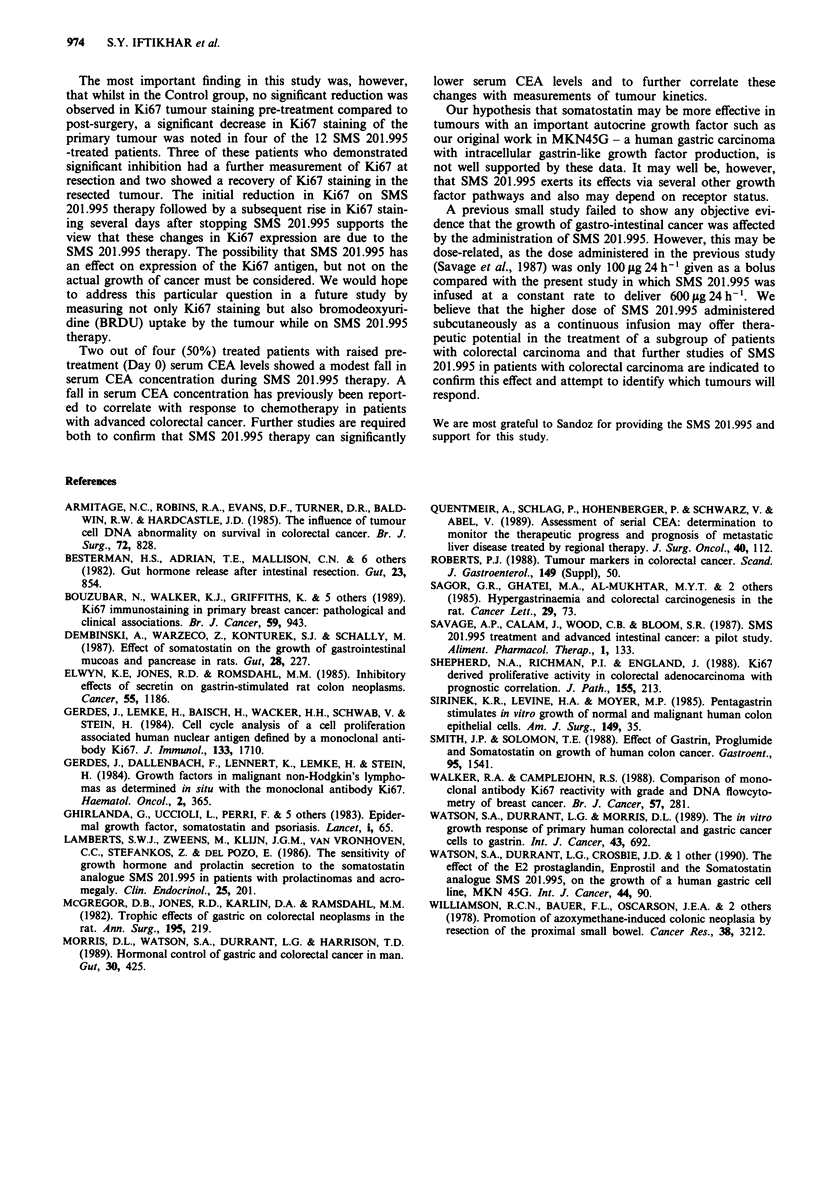# The effect of long acting somatostatin analogue SMS 201.995 therapy on tumour kinetic measurements and serum tumour marker concentrations in primary rectal cancer.

**DOI:** 10.1038/bjc.1991.212

**Published:** 1991-06

**Authors:** S. Y. Iftikhar, S. A. Watson, D. L. Morris

**Affiliations:** Department of Surgery, University Hospital, Nottingham, UK.

## Abstract

Twelve patients with rectal carcinoma were treated for 2 weeks with the somatostatin analogue SMS 201.995. Effects of this therapy were assessed using serum marker concentration, Ki67 and gastrin-immunoreactivity of the primary tumour. In four out of 12 patients, a significant decrease in Ki67 immunoreactivity was seen during SMS 201.995 treatment while in the remaining eight patients there was no significant change in Ki67 expression. Four patients had elevated pretreatment serum carcinoembryonic antigen (CEA) levels. In two of these four patients, serum CEA levels fell modestly during SMS 201.995 therapy. This is the first clinical evidence that a somatostatin analogue can inhibit the growth of some colorectal cancers.


Br. J. Cancer (1991), 63, 971–974 © Macmillan Press Ltd., 1991

### The effect of long acting somatostatin analogue SMS 201.995 therapy on tumour kinetic measurements and serum tumour marker concentrations in primary rectal cancer

S.Y. Iftikhar[1], S.A. Watson[2] & D.L. Morris[3]

[1]*Senior Registrar, Department of Surgery, University Hospital, Nottingham;* [2]*Research Scientist, Cancer Research Campaign Laboratories, Nottingham, UK;* [3]*Professor of Surgery, St George Hospital, Sydney, NSW, Australia.*

**Summary** Twelve patients with rectal carcinoma were treated for 2 weeks with the somatostatin analogue SMS 201.995. Effects of this therapy were assessed using serum marker concentration, Ki67 and gastrin-immunoreactivity of the primary tumour. In four out of 12 patients, a significant decrease in Ki67 immunoreactivity was seen during SMS 201.995 treatment while in the remaining eight patients there was no significant change in Ki67 expression. Four patients had elevated pretreatment serum carcinoembryonic antigen (CEA) levels. In two of these four patients, serum CEA levels fell modestly during SMS 201.995 therapy. This is the first clinical evidence that a somatostatin analogue can inhibit the growth of some colorectal cancers.

Colorectal cancer is the second commonest malignancy in England and Wales, responsible for 23,500 new cases and over 17,300 deaths annually. In cases where the tumour is not cured by surgery or where advanced disease is diagnosed, the only therapies currently available are radiotherapy and chemotherapy. Whilst both modalities have achieved real benefit for patients with colon cancer, their contributions are modest and should not discourage us from looking for novel methods of growth control.

Gastrin is an established growth factor for human gastric and colorectal cancers (Morris *et al.*, 1989; Watson *et al.*, 1989). The peptide hormone, somatostatin, has been found to reduce circulating levels of gastrin and reduces the growth of human colorectal xenografts growing in experimental animals (Smith *et al.*, 1988). The long acting somatostatin analogue, SMS 201.995, has also been shown to inhibit the growth of human gastric cancer xenografts (Watson *et al.*, 1990). In addition, SMS 201.995 has been shown to reduce serum concentrations of other potential tumour growth factors such as insulin-like growth factor (IGF 1) and epidermal growth factor (EGF) (Ghirlanda *et al.*, 1983; Lambert *et al.*, 1986).

CEA levels are elevated in the serum of a significant number of patients with advanced colorectal cancer (Roberts *et al.*, 1988). Changes in sequential serum CEA levels have been reported to correlate with response to chemotherapy in patients with metastatic disease (Quentmeir *et al.*, 1989). Sequential serum CEA levels may therefore be of value in monitoring response to systemic endocrine therapy such as the somatostatin analogue SMS 201.995.

The monoclonal antibody Ki67 reacts with a nuclear antigen reported to be present in actively dividing cells (i.e., G1, S, $G_2$ and M phases of the cell cycle) but not in quiescent (GO) cells (Gerdes *et al.*, 1984; Gerdes *et al.*, 1984) in breast cancer Ki67 antigen expression correlates significantly with histological grade and mitotic index of the primary tumour (Walker *et al.*, 1988; Bouzubar *et al.*, 1989). Ki67 antigen expression has also been reported to correlate significantly with mitotic index in primary colorectal tumours (Shepherd *et al.*, 1988). Sequential Ki67 measurements of the primary tumour may therefore be a further method of assessment of response to endocrine therapy in rectal carcinoma.

The aim of the present study is to evaluate the effect of subcutaneously administered SMS 201.995 on both the change in serum tumour marker concentrations and in tumour kinetics, as assessed by Ki67 immunoreactivity, in patients with primary rectal carcinoma

Correspondence: D.L. Morris.
Received 28 March 1990; and in revised form 23 January 1991.

### Materials and methods

### *Patient details*

From January 1989 to October 1989, 19 patients with histologically confirmed primary rectal adenocarcinoma were considered suitable for entry into this study. Patients with a history of myocardial infarction within 6 months prior to the study, liver or renal failure and any patients who had received any investigational drug within 4 weeks of entry, were excluded. Of the 19 eligible patients entered into this study, 13 were treated with SMS 201.995 (Sandoz), and six patients were untreated controls. Treatment/control was not allocated randomly. The controls were patients who did not wish to be included in the somatostatin study but consented to the biopsies. This study was approved by the ethical committee of University Hospital, Nottingham. All patients gave informed consent and were able to comply with the study protocol. One insulin-dependent diabetic patient was withdrawn on the 4th day of SMS 201.995 therapy because of recurrent hypoglycaemia due to unstable diabetes. Twelve patients are therefore assessable for response to SMS 201.995.

### *Treatment schedule*

SMS 201.995 was administered subcutaneously for 14 days by continuous infusion at a dose of $600\,\mu g\,24\,h^{-1}$ with an autosyringe and pump. Fasting blood samples were taken prior to initiation of SMS 201.995 therapy (Day 0), on the 7th day of treatment and at the termination of treatment: full blood count, urea and electrolytes, liver function tests, blood sugar, serum gastrin and serum concentration of the tumour markers CA 19.9 and carcinoembryonic antigen (CEA) were measured. Measurement of both tumour markers was performed in our own laboratory using the commercially available CIS radioimmunoassay kits. Serum gastrin was regarded as elevated if it was above $110\,ng\,l^{-1}$ (normal range 25–110 $ng\,l^{-1}$). Similarly, serum CEA and CA 19.9 concentrations were regarded as elevated if they were above $10\,ng\,ml^{-1}$ and 33 units $ml^{-1}$ respectively.

### *Tumour sampling*

Multiple rectal tumour biopsies were taken at initiation and termination of SMS 201.995 therapy from the same areas of the tumour under direct vision at rigid sigmoidoscopy. The multiple biopsies of the tumour mass were pooled to yield as representative a sample as possible. Following 14 days of SMS 201.995 therapy, the primary tumour was resected at which time further biopsies of the tumour were taken. In control patients, samples were taken at sigmoidoscopy and then again at the time of resection.

### *Measurement of tumour proliferation with Ki67 monoclonal antibody*

Tumour biopsy material was disaggregated enzymatically with collagenase to achieve a single cell suspension (4). The cell suspension was then fixed (10 min, 1% paraformaldehyde 4°C, followed by 10 min 70% ethanol, at 4°C). One hundred µl of the mouse monoclonal antibody, Ki67 (Dakopatts, Bucks, UK) at a 1/10 dilution was added to $2 \times 10^5$ cells and incubated for 5 min at 37°C followed by 55 min at room temperature (22°C). The cells were washed and antibody binding was detected with an anti-mouse fluoroscein isothiocyanate (FITC) conjugate (Dakopatts) used at a 1/80 dilution (0.5 ml/$2 \times 10^5$ cells) with an incubation period of 30 min at room temperature. Cells were analysed on a fluorescence-activated cell sorter (Armitage *et al.*, 1985) and the mean linear fluorescence/cell and percentage positive cells/population were analysed. Debris and small cells such as RBC and lymphocytes were gated out of the analysis.

### *Measurements of intracellular gastrin immunoreactivity*

The single cell suspension from the tumour biopsy specimens were also analysed for gastrin immunoreactivity by flow cytometric analysis. One thousand µl of rabbit anti-human gastrin (G17) antiserum (Dakopatts) at 1/100 dilution (46 ng protein$^{-1}$ ml$^{-1}$) was added to $2 \times 10^5$ cells, and incubated at room temperature for 60 min. The cells were washed and an anti-rabbit FITC conjugate (Dakopatts) at 1/80 dilution (0.5 ml/$2 \times 10^5$ cells) then added. The degree of gastrin immunoreactivity was assessed by flow cytometry. Specificity of anti-gastrin binding was determined by preabsorbing the antiserum for 3 h at 4°C with 50 µg ml$^{-1}$ human G17 (Sigma, Dorset, UK). Any staining after preabsorption was regarded as non-specific binding.

### *Statistical analysis*

Pooled tumour tissue was examined in duplicate for both Ki67 and gastrin immunoreactivity. These data for pretreatment or during treatment were compared by the student *t*-test. Triplicate samples of blood were analysed for tumour markers.

### Results

### *Serum markers*

Of the six Control patients who did not receive SMS 201.995, one had an elevated CEA concentration which did not alter pre-operatively (Figure 1a).

Of 12 patients who completed the course of SMS 201.995 therapy, four had elevated CEA levels (Figure 1b). All four patients had Dukes C carcinomas. None had liver metastases either clinically or on intra-operative contact ultrasonography. In two of these four patients, there was a modest reduction in serum CEA levels during SMS 201.995 therapy. In all four patients, post-resection serum CEA levels fell below pre-operative levels, as would be expected.

Serum CA 19.9 concentrations in Control and SMS 201.995 treated patients were similar both pre- or post-operatively. Serum gastrin concentration remained within the normal range in both treated and untreated patients (Figures 2a and b), the normal range of serum gastrin being 25–110 ng l$^{-1}$.

### *Ki67 Immunoreactivity*

In the six Control patients, tumour biopsies for Ki67 measurements were obtained pre-operatively on Day 0 and again from the resected tumour specimens. In five of six patients, the Ki67 measurements of the pre-treatment (Day 0) biopsies were not significantly different from tumour specimens (Table I). In one patient, the pre-treatment (Day 0) biopsy gave a lower value for Ki67 staining than the post-operative tumour specimen (Table I).

In contrast, four of the 12 SMS 201.995 treated patients (Patients 1, 2, 5 and 6; Table II) had significantly lower Ki67 immunostaining in the post-SMS 201.995 treated (Day 14) tumour biopsy compared to the pre-treatment (Day 0) biopsy (Table II) ($P < 0.05$). Ki67 staining remained at this significantly lower level post-operatively in one of the four tumours. In two of the remaining tumours operated on 10 and 23 days after stopping SMS 201.995, there was a significant recovery in Ki67 staining of the tumour in material resected at operation (Table II). The fourth patient with a significant decrease in Ki67 during therapy did not have a post-treatment measurement due to an error in tissue handling.

**Figure 1** **a**, Serum CEA profile in primary rectal carcinoma (control group). **b**, Serum CEA profile in primary rectal carcinoma patients treated with SMS 201.995.

**Figure 2** **a**, Fasting serum gastrin in control group; **b**, Fasting serum gastrin profile in SMS 201.995 treatment patients with primary rectal carcinoma.

*Gastrin immunoreactivity*

Anti-gastrin activity was measured in the tumours of only two Control patients. One patient showed a significant elevation in the % of tumour cells staining positively in the post-operative tumour specimen compared to the pre-treatment (Day 0) biopsy (Table III). In the SMS 201.995 group, two patients, Patients 2 and 6 (Table III), not only had reduced Ki67 staining post-SMS 201.995 therapy, but also had significantly reduced anti-gastrin staining. In Patient 2, there was a 'rebound' in gastrin staining of the post-operative tumour specimen resected 10 days after cessation of therapy, which reflected the rebound rise observed in this same patient with Ki67 staining. Patient 8 (Table II) also has a significant reduction in anti-gastrin immunoreactivity following SMS 201.995 therapy: Ki67 immunoreactivity was low in both pre- and post-SMS 201.995 tumour biopsies. In one treated patient (Patient 2, Table III), the pre-treatment (Day 0) biopsy showed significantly lower anti-gastrin staining than measured in both the post-treatment (Day 0) biopsy and the post-operative tumour specimens.

**Table I** % Tumour cells (±s.d.) staining positive ( + ) for Ki67 in control patients

| Patient no. | *Pre-treatment biopsy* *% ( + ) cells* | *s.d.* | *Resected tumour* *% ( + ) cells* | *s.d.* | |
|---|---|---|---|---|---|
| 1 | <2 | | 8.4 | (0.2) | |
| 2 | 4.1 | (0.3) | 5.4 | (1) | NS |
| 3 | 2.4 | (0.1) | 6.5 | (3) | NS |
| 4 | 12.2 | (3) | 15.4 | (0.5) | NS |
| 5 | 12.2 | (5) | 12.2 | | |
| 6 | 3.4 | (0.1) | 2.1 | (1) | NS |

% ( + ) cells is calculated as mean of duplicate samples.

### Discussion

Hypergastrinaemia has been implicated in the increased growth of colon cancer in animal models who had proximal resection of the small bowel (Williamson *et al.*, 1978) and antral exclusion (McGregor *et al.*, 1982; Besterman *et al.*, 1982; Elwyn *et al.*, 1985; Sagor *et al.*, 1985). In this study, SMS 201.995 failed to significantly effect serum gastrin levels (Figures 2a and b). However, it could still be possible that SMS 201.995 may affect the local production of gastrin by the tumour in the same way that it prevents gastrin release by antral cells (Dembinski *et al.*, 1987), thereby blocking an autocrine/paracrine growth pathway (Sirinek *et al.*, 1985). This was addressed in our study by studying intracellular tumour immunoreactivity to gastrin.

We have previously shown that SMS 201.995 therapy reduced the growth of a human gastric adenocarcinoma xenograft grown in nude mice, the cell line used being known to have high level of gastrin immunoreactivity (Watson *et al.*, 1990). In this study, SMS 201.995 reduced gastrin immunoreactivity in the primary tumour in three of the 12 SMS 201.995 treated patients: in two of these three patients, a parallel decrease in Ki67 staining was also noted. However, it should be noted that in three patients there was an elevation of intracellular tumour gastrin during therapy (Table II) which was not associated with any increase in Ki67 immunoreactivity (Table II).

**Table II** % Tumour cells (±s.d.) staining positive ( + ) for Ki67 in SMS 201.995 treated patients

| Patient no. | *Pre-treatment biopsy* *% ( + ) cells* | *s.d.* | *Post-treatment biopsy* *% ( + ) cells* | *s.d.* | *Resected tumour* *% ( + ) cells* | *s.d.* | *Time from cessation of treatment to tumour resection (Days)* |
|---|---|---|---|---|---|---|---|
| 1 | 5.0 | (0.4) | <2 | (0.2)[a] | 12.0 | (1)[a] | 10 |
| 2 | 6.5 | (0.7) | <2 | (1)[b] | <2 | (0.4)[c] | 13 |
| 3 | 5.5 | (0.1) | n.d. | n.d. | 8.0 | (0.5) | 1 |
| 4 | 5.4 | (4) | 3.8 | (1.6) | <2 | (0.5) | 1 |
| 5 | 8.1 | (0.7) | <2 | (2.6[b]) | n.d. | n.d. | n.d. |
| 6 | 7.7 | (0.7) | <2 | (0.5)[c] | 5.9 | | 23 |
| 7 | 4.1 | (0.1) | n.d. | n.d. | 4.9 | (0.2) | 1 |
| 8 | <1 | (0.3) | <2 | (0.8) | n.d. | n.d. | 1 |
| 9 | <2 | (0.35) | <2 | (0.2) | n.d. | n.d. | 7 |
| 10 | 12.4 | (0.2) | 23 | (8.5) | 15.3 | (1) | 1 |
| 11 | <2 | (0.5) | 4.2 | (1) | 5.3 | (1) | 6 |
| 12 | <2 | (0.15) | 4.1 | (2) | 3.1 | (2) | 6 |

[a]$P<0.01$; [b]$P<0.05$; [c]$P<0.02$; When compared to staining achieved with pre-treatment biopsy. n.d. – not determined. % ( + ) cells is calculated as mean of duplicate samples.

**Table III** % Tumour cells (±s.d.) staining positive ( + ) for anti-G17 immunoreactivity in control and SMS 201.995 treated patients

| Patient no. | *Pre-treatment biopsy* *% ( + ) cells* | *s.d.* | *Post-treatment biopsy* *% ( + ) cells* | *s.d.* | *Resected tumour* *% ( + ) cells* | *s.d.* |
|---|---|---|---|---|---|---|
| Control 2 | 8.3 | (0.1) | n.d. | n.d. | 15.4 | (1.1)[a] |
| Control 6 | 4.9 | (1.4) | n.d. | n.d. | 1.2 | (0.5) |
| SMS treated 1 | 4.2 | (0.9) | <2 | (0.25)[b] | 22 | (7.5)[b] |
| SMS treated 2 | <2 | (0.5) | 11 | (1)[c] | 9 | (0.8)[c] |
| SMS treated 4 | <2 | | n.d. | n.d. | 9.3 | (1.2) |
| SMS treated 5 | <2 | | <2 | | n.d. | n.d. |
| SMS treated 6 | 14 | (0.3) | <2 | (0.1)[d] | n.d. | n.d. |
| SMS treated 7 | <2 | (0.1) | n.d. | n.d. | 6.7 | (0.5) |
| SMS treated 8 | 69.3 | (9.9) | 3.8 | (0.4)[a] | n.d. | n.d. |
| SMS treated 9 | 9.5 | | 14 | (0.1) | n.d. | n.d. |
| SMS treated 11 | 2 | (0.4) | 5.7 | (2.1) | 2.1 | |
| SMS treated 12 | <2 | (0.3) | <2 | | n.d. | n.d. |

[a]$P<0.01$; [b]$P<0.05$; [c]$P<0.002$; [d]$P<0.001$, when compared to staining achieved with pre-treatment biopsy. n.d. – not determined. % ( + ) cells is calculated as mean of duplicate samples.

The most important finding in this study was, however, that whilst in the Control group, no significant reduction was observed in Ki67 tumour staining pre-treatment compared to post-surgery, a significant decrease in Ki67 staining of the primary tumour was noted in four of the 12 SMS 201.995 -treated patients. Three of these patients who demonstrated significant inhibition had a further measurement of Ki67 at resection and two showed a recovery of Ki67 staining in the resected tumour. The initial reduction in Ki67 on SMS 201.995 therapy followed by a subsequent rise in Ki67 staining several days after stopping SMS 201.995 supports the view that these changes in Ki67 expression are due to the SMS 201.995 therapy. The possibility that SMS 201.995 has an effect on expression of the Ki67 antigen, but not on the actual growth of cancer must be considered. We would hope to address this particular question in a future study by measuring not only Ki67 staining but also bromodeoxyuridine (BRDU) uptake by the tumour while on SMS 201.995 therapy.

Two out of four (50%) treated patients with raised pre-treatment (Day 0) serum CEA levels showed a modest fall in serum CEA concentration during SMS 201.995 therapy. A fall in serum CEA concentration has previously been reported to correlate with response to chemotherapy in patients with advanced colorectal cancer. Further studies are required both to confirm that SMS 201.995 therapy can significantly lower serum CEA levels and to further correlate these changes with measurements of tumour kinetics.

Our hypothesis that somatostatin may be more effective in tumours with an important autocrine growth factor such as our original work in MKN45G – a human gastric carcinoma with intracellular gastrin-like growth factor production, is not well supported by these data. It may well be, however, that SMS 201.995 exerts its effects via several other growth factor pathways and also may depend on receptor status.

A previous small study failed to show any objective evidence that the growth of gastro-intestinal cancer was affected by the administration of SMS 201.995. However, this may be dose-related, as the dose administered in the previous study (Savage *et al.*, 1987) was only $100\,\mu g\,24\,h^{-1}$ given as a bolus compared with the present study in which SMS 201.995 was infused at a constant rate to deliver $600\,\mu g\,24\,h^{-1}$. We believe that the higher dose of SMS 201.995 administered subcutaneously as a continuous infusion may offer therapeutic potential in the treatment of a subgroup of patients with colorectal carcinoma and that further studies of SMS 201.995 in patients with colorectal carcinoma are indicated to confirm this effect and attempt to identify which tumours will respond.

We are most grateful to Sandoz for providing the SMS 201.995 and support for this study.

### References

ARMITAGE, N.C., ROBINS, R.A., EVANS, D.F., TURNER, D.R., BALDWIN, R.W. & HARDCASTLE, J.D. (1985). The influence of tumour cell DNA abnormality on survival in colorectal cancer. *Br. J. Surg.*, **72**, 828.

BESTERMAN, H.S., ADRIAN, T.E., MALLISON, C.N. & 6 others (1982). Gut hormone release after intestinal resection. *Gut*, **23**, 854.

BOUZUBAR, N., WALKER, K.J., GRIFFITHS, K. & 5 others (1989). Ki67 immunostaining in primary breast cancer: pathological and clinical associations. *Br. J. Cancer*, **59**, 943.

DEMBINSKI, A., WARZECO, Z., KONTUREK, S.J. & SCHALLY, M. (1987). Effect of somatostatin on the growth of gastrointestinal mucoas and pancrease in rats. *Gut*, **28**, 227.

ELWYN, K.E, JONES, R.D. & ROMSDAHL, M.M. (1985). Inhibitory effects of secretin on gastrin-stimulated rat colon neoplasms. *Cancer*, **55**, 1186.

GERDES, J., LEMKE, H., BAISCH, H., WACKER, H.H., SCHWAB, V. & STEIN, H. (1984). Cell cycle analysis of a cell proliferation associated human nuclear antigen defined by a monoclonal antibody Ki67. *J. Immunol.*, **133**, 1710.

GERDES, J., DALLENBACH, F., LENNERT, K., LEMKE, H. & STEIN, H. (1984). Growth factors in malignant non-Hodgkin's lymphomas as determined *in situ* with the monoclonal antibody Ki67. *Haematol. Oncol.*, **2**, 365.

GHIRLANDA, G., UCCIOLI, L., PERRI, F. & 5 others (1983). Epidermal growth factor, somatostatin and psoriasis. *Lancet*, **i**, 65.

LAMBERTS, S.W.J., ZWEENS, M., KLIJN, J.G.M., VAN VRONHOVEN, C.C., STEFANKOS, Z. & DEL POZO, E. (1986). The sensitivity of growth hormone and prolactin secretion to the somatostatin analogue SMS 201.995 in patients with prolactinomas and acromegaly. *Clin. Endocrinol.*, **25**, 201.

MCGREGOR, D.B., JONES, R.D., KARLIN, D.A. & RAMSDAHL, M.M. (1982). Trophic effects of gastric on colorectal neoplasms in the rat. *Ann. Surg.*, **195**, 219.

MORRIS, D.L., WATSON, S.A., DURRANT, L.G. & HARRISON, T.D. (1989). Hormonal control of gastric and colorectal cancer in man. *Gut*, **30**, 425.

QUENTMEIR, A., SCHLAG, P., HOHENBERGER, P. & SCHWARZ, V. & ABEL, V. (1989). Assessment of serial CEA: determination to monitor the therapeutic progress and prognosis of metastatic liver disease treated by regional therapy. *J. Surg. Oncol.*, **40**, 112.

ROBERTS, P.J. (1988). Tumour markers in colorectal cancer. *Scand. J. Gastroenterol.*, **149** (Suppl), 50.

SAGOR, G.R., GHATEI, M.A., AL-MUKHTAR, M.Y.T. & 2 others (1985). Hypergastrinaemia and colorectal carcinogenesis in the rat. *Cancer Lett.*, **29**, 73.

SAVAGE, A.P., CALAM, J., WOOD, C.B. & BLOOM, S.R. (1987). SMS 201.995 treatment and advanced intestinal cancer: a pilot study. *Aliment. Pharmacol. Therap.*, **1**, 133.

SHEPHERD, N.A., RICHMAN, P.I. & ENGLAND, J. (1988). Ki67 derived proliferative activity in colorectal adenocarcinoma with prognostic correlation. *J. Path.*, **155**, 213.

SIRINEK, K.R., LEVINE, H.A. & MOYER, M.P. (1985). Pentagastrin stimulates *in vitro* growth of normal and malignant human colon epithelial cells. *Am. J. Surg.*, **149**, 35.

SMITH, J.P. & SOLOMON, T.E. (1988). Effect of Gastrin, Proglumide and Somatostatin on growth of human colon cancer. *Gastroent.*, **95**, 1541.

WALKER, R.A. & CAMPLEJOHN, R.S. (1988). Comparison of monoclonal antibody Ki67 reactivity with grade and DNA flowcytometry of breast cancer. *Br. J. Cancer*, **57**, 281.

WATSON, S.A., DURRANT, L.G. & MORRIS, D.L. (1989). The *in vitro* growth response of primary human colorectal and gastric cancer cells to gastrin. *Int. J. Cancer*, **43**, 692.

WATSON, S.A., DURRANT, L.G., CROSBIE, J.D. & 1 other (1990). The effect of the E2 prostaglandin, Enprostil and the Somatostatin analogue SMS 201.995, on the growth of a human gastric cell line, MKN 45G. *Int. J. Cancer*, **44**, 90.

WILLIAMSON, R.C.N., BAUER, F.L., OSCARSON, J.E.A. & 2 others (1978). Promotion of azoxymethane-induced colonic neoplasia by resection of the proximal small bowel. *Cancer Res.*, **38**, 3212.